# Synthesis of Polymer Nanocomposites Based on [Methyl Cellulose]_(1−*x*)_:(CuS)_x_ (0.02 M ≤ *x* ≤ 0.08 M) with Desired Optical Band Gaps

**DOI:** 10.3390/polym9060194

**Published:** 2017-05-30

**Authors:** Shujahadeen B. Aziz, Mariwan A. Rasheed, Hameed M. Ahmed

**Affiliations:** 1 Advanced Materials Research Lab., School of Science-Department of Physics, Faculty of Science and Science Education, University of Sulaimani, Qlyasan Street, Sulaymaniyah 46001, Kurdistan Regional Government, Iraq; hameed.ahmed@univsul.edu.iq; 2 DevelopmentCenter for Research and Training (DCRT), University of Human Development, Qrga Street, Sulaymaniyah 46001, Kurdistan Regional Government, Iraq; mariwan.rasheed@uhd.edu.iq

**Keywords:** methyl cellulose (MC) nanocomposite, SPR band, bandgap study, optical dielectric function, XRD, FTIR study

## Abstract

In this paper, the sample preparation of polymer nanocomposites based on methyl cellulose (MC) with small optical bandgaps has been discussed. Copper monosulfide (CuS) nanoparticles have been synthesized from the sodium sulphide (Na_2_S) and copper chloride (CuCl_2_) salts. Distinguishable localized surface resonance plasmon (LSRP) absorption peaks for CuS nanoparticles within the 680–1090 nm scanned wavelength range were observed for the samples. An absorption edge (*E_d_*) was found to be widely shifted to a lower photon energy region. A linear relationship between the refractive index of the samples and the CuS fraction was utilized to describe the distribution of the particle. The optical bandgap of MC was reduced from 6.2 to 2.3 eV upon the incorporation of 0.08 M of CuS nanoparticles. The optical dielectric loss, as an alternative method, was used successfully to estimate the optical bandgap. Moreover, the electronic transition type was identified by using Tauc’s extrapolation method. The plots of the optical dielectric constant and energy bandgap as a function of the CuS concentration were utilized to examine the validity of the Penn model. For the nanocomposite samples, the Urbach energy was found to be increased, which can be evidence for a large possible number of bands-to-tail and tail-to-tail transitions. However, from the X-ray diffraction (XRD) analysis, it was also found that the synthesized CuS nanoparticles disrupted the crystallinity phase of the MC polymer. Finally, fourier transform infrared (FTIR) spectroscopy for the samples was also performed. Significant decreases of transmittance intensity as well as band shifting in the FTIR spectra were observed for the doped samples.

## 1. Introduction

Recently, semiconductor nanostructures are broadly used in photovoltaic devices, such as dye sensitized, all-inorganic nanoparticles, and hybrid nanocrystal/polymer solar cells [1]. Metal sulfide nanoparticles, such as copper sulfides, have excellent optoelectronic properties. They are particularly interesting due to their ability of forming various stoichiometries [2]. Previous studies revealed that metal copper sulfide nanoparticles have numerous stable and metastable types, such as Cu2S (Chalcocite) and CuS (Covellite) compositions [3]. It is established that the CuS is the more stable composition compared to the other copper sulfides [2,3]. On the other hand, cellulose is the most abundant organic polymer in nature and, accordingly, very favorable as a renewable resource [4]. Cellulose is a natural polymer and it is considered as an alternative organic material which may replace the petrochemical polymers [5]. One of the most abundant, inexpensive, and environmentally friendly classes is Methyl cellulose (MC). It is one of the types of modified cellulose, which can be considered as an alternative to synthetic polymers [6].The good mechanical, thermal, and chemical stabilities, as well as the excellent properties of film-forming and solubility are among those properties that make the MC exceptional for this purpose [7]. MC is an amorphous polymer with a high glass transition temperature (*T*_g_), ranging from 184 to 200 °C. MC has non-bonding pairs of electron at its oxygen atoms, which can provide complexation sites for the cation salt to be bonded [8]. For the last two decades, polymer nanocomposites have attracted great attention both scientifically and technologically [9]. Polymer nanocomposites containing semiconductor nanoparticles are shown to be significant for building electronic and optoelectronic devices, such as solar cells, light-emitting diodes, optical limiters, and various types of sensors [10]. The attention of many material scientists has been drawn to the study of fabrication and development of polymer-based nanocomposites for their potential applications in technology [11]. However, the drawbacks of the use of inorganic nanostructured materials can be controlled by embedding a relatively small content of inorganic nanoparticles within the polymer matrix [9]. Finding a semiconductor material with a suitable bandgap of around 1 eV can be one of the most challenging points in the area of photovoltaic and optoelectronics [1]. The technological importance of such materials can be directly associated to understanding the relationship between the optical properties and structural characteristics of the nanocomposite. This, therefore, leads to the prediction of its suitability in various applications [12]. It was reported that most of conjugated polymers, that have an intramolecular charge transfer (ICT) property, are narrow bandgap materials [13]. Even the incorporation of both donor and acceptor type semiconductors into conjugated polymers may be a good approach to fabricate photovoltaic devices with good external quantum efficiency [14,15]. However, the drawbacks such as low efficiency and fast degradation are the common problems of conjugated polymers [16]. On the other hand, polar polymers such as MC, Poly(vinyl alcohol) (PVA), and chitosan are low cost, have long lifetimes, and they have good film-forming ability. From this view point, our group worked hard to prepare polymer nanocomposites based on polar polymers with the desired optical band gaps. The main objective of this work is to synthesize MC based nanocomposites with good film forming ability, transparency, and a small optical bandgap, using in situ techniques.

## 2. Experimental Details

### 2.1. Materials and Sample Preparation

**Scheme 1:**
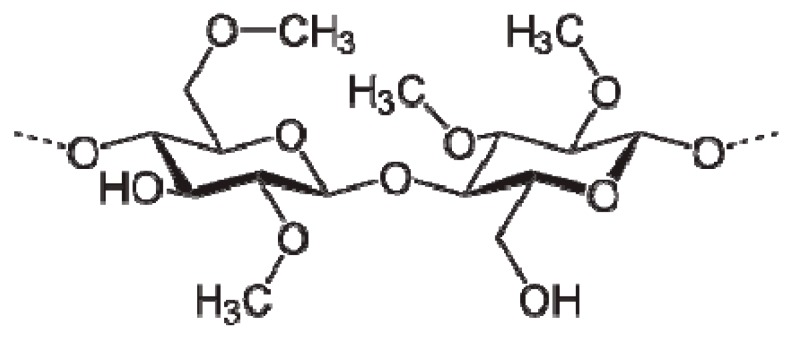
Repeating unit of methylcellulose.

Solid polymer nanocomposites based on MC:*_x_*CuS (0.02 M ≤ *x* ≤ 0.08 M) were prepared by the solution cast technique at room temperature. The MC powder was first dissolved in distilled water. The solution was then well stirred by a magnetic stirrer for 24 h to obtain a homogenous solution. Sodium sulphide (Na_2_S) and copper chloride (CuCl_2_) salts were used for the synthesis of CuS nanoparticles. They were separately dissolved in 5 mL distilled water at room temperature. Under continuous stirring, different molar concentrations of CuCl_2_ salt were individually added to the MC solution. Subsequently, equal amounts of Na_2_S salt were slowly added to the solutions. The in situ preparation method for the polymer nanocomposite is significant due to the fact that the host polymer (MC) can act as capping and stabilizing agents for the CuS nanoparticles due to the existence of OH groups on the MC backbone structure. Consequently, this avoids the aggregation and precipitation of the CuS nanoparticles. The repeating units of methylcellulose are shown in Scheme 1. 

The content of CuS nanoparticles in the prepared samples was varied from 0.02 to 0.08 M in steps of 0.02 M in the volume fraction. Furthermore, the mixtures were continuously stirred until homogeneous solutions were achieved. The samples were then coded as MCPN0, MCPN1, MCPN2, MCPN3, and MCPN4 for MC doped with 0, 0.02, 0.04, 0.06, and 0.08 M of CuS, respectively. The color of the composite samples turned to green due to the formation of CuS nanoparticles. The intense green color was evidence for further formation of CuS nanoparticles. Different plastic Petri dishes were used for the homogenous solutions to be casted. They were then allowed to be slowly evaporated at room temperature for three weeks until homogenous films were achieved. Finally, for further drying, the films were kept in desiccators with blue silica gel desiccant. 

### 2.2. Ultraviolet-Visible (UV-Vis) Spectroscopy

The ultraviolet-visible (UV-vis) spectra for pure Methyl cellulose (MC) and MC nanocomposite films were measured, using a double beam ultraviolet-visible-near infrared (UV-Vis-NIR) spectrophotometer (Perkin Elmer, Lambda 25, Waltham, MA, USA).

### 2.3. Fourier Transform Infrared (FTIR) and X-ray Diffraction (XRD) Spectroscopies

A FTIR spectrophotometer (Thermo Scientific, Nicolet iS10,(Perkin Elmer, Waltham, MA, USA), in the wavenumber region 4000–400 cm^−1^ with a resolution of 2 cm^−1^, was used to analyze the pure MC and doped MC samples. All the samples were also analyzed by a monochromatic X-ray beam with wavelength λ = 1.5406 A° and 2θ ranged from 10° ≤ 2θ ≤ 70° with a 0.1° step size.

## 3. Results and Discussion

### 3.1. Optical Properties

#### 3.1.1. Transmittance and Absorption Study

**Figure 1:**
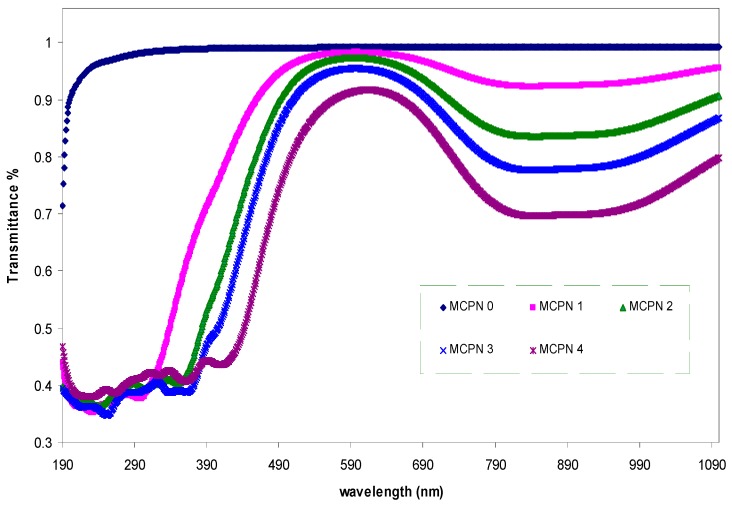
The transmittance spectra of pure MC (methyl cellulose) and doped MC samples.

Figure 1 shows the transmittance of pure MC and doped MC with different CuS concentrations. From the figure, it is shown that a high transparency over 97% was found above the visible region for the pure MC sample, while below this region the transparency was lower. However, it is clear that the transparency was found to be decreased with increasing CuS concentration. The highest doping samples showed the lowest transparency. This is due to the high refractive index and the effect of scattering in the highly doped films [17]. The requirement of maintaining transparency is found to be one of the challenges of creating nanocomposite materials for optical applications [18]. Interestingly, the transparency of the doped samples was found to be above 80% in the visible region. Here, the doped samples’ spectra show a shoulder shape while such features do not exist for the pure MC. This is because the CuS nanoparticles’ formation caused the transparency decrease. The embedded CuS nanoparticles are responsible for the high refractive index and the scattering in the samples. This will be discussed more in the later sections.

**Figure 2:**
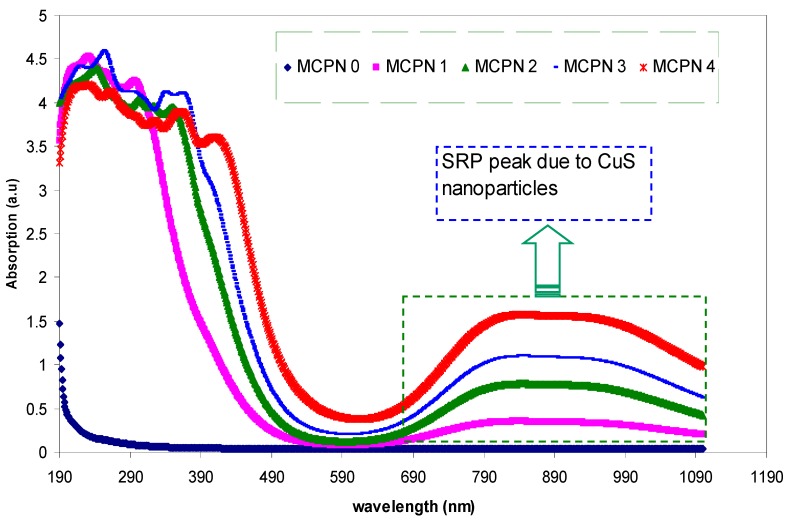
The absorption spectra of pure MC and MC nano-composite samples.

The absorption spectra for the pure and doped MC samples were also recorded, as shown in Figure 2. Here, an absorption band near the infrared (IR) region was seen for the doped samples while in the absorption spectrum of the pure MC sample, it was found to be absent. Previous studies confirmed that from the UV-Vis absorption spectrum of the Cu_2_S nanocrystals, a wide absorption up to approximately 1000 nm without any appearing IR bands can be observed [1,2,3]. The nature of the IR band was found to be a fundamental problem in discussing the difference between the distinct types of Cu_x_S samples. It was noted that the appearance of the IR band is a confirmation of the formation of the green crystalline CuS nanoparticles [2]. Therefore, the formation of CuS nanoparticles is verified by the appearance of the strong band absorption at 890 nm. This can be attributed to the well-known localized surface plasmon resonance (LSPR) phenomenon. The disappearance of the clear shoulders at 450 nm, which is a predictable absorption peak of the Cu_2_S phase, reveals the successful CuS nanoparticles’ fabrication [18,19]. The strong absorption in the range of visible wavelengths (690 to 1000 nm) can be attributed to the LSPR, which originates from the collective oscillation of the free conduction electrons in reply to the electromagnetic waves [20]. The local electromagnetic fields at the nanoparticle surface can be manipulated and enhanced due to the plasmon resonance [21]. The resonant photon wavelength changes for different metals [17,22]. According to previous reports [23,24,25], the position of the LSPR band can be manipulated through controlling the concentration, size, shape, and behavior of the metal nanoparticles, as well as the dielectric behavior of the host materials. It is obvious that the LSPR peaks are broader rather than sharper. This could be due to the larger size distribution of the nanoparticles and their neighboring effects [20].

**Figure 3:**
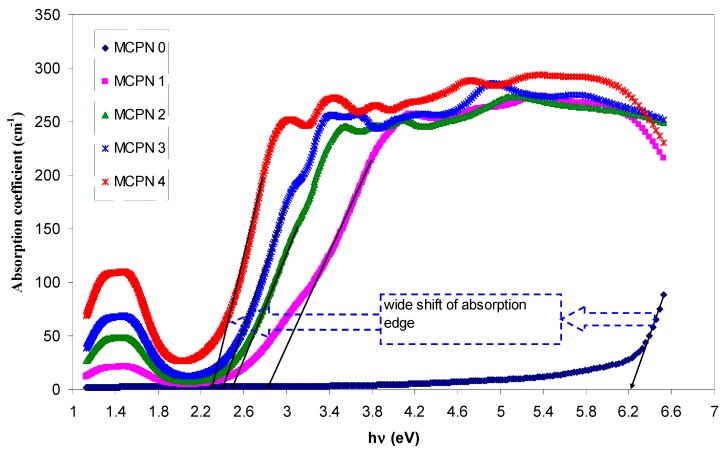
The absorption coefficient spectra against *hυ* for pure MC and doped MC films.

It is believed that the fundamental absorption edge is one of the most significant characteristics of the absorption spectra of crystalline and non-crystalline (or amorphous) materials [26]. The optical absorption coefficient (α) as a function of the wavelength (λ) of the pure MC and MC nanocomposite films can be evaluated using the following relation [27]:

(1) $$\alpha =\frac{1}{d}\ln\left[\frac{{\left(1-R\right)}^{2}}{2T}+\sqrt{\frac{{\left(1-R\right)}^{4}}{4T^{2}}+R^{2}}\right]$$

where *d* is the thickness of the films. The optical absorption coefficient as a function of photon energy was determined for the pure and doped MC samples, as shown in Figure 3. It is obvious that the absorption edge has been shifted to the lower photon energy with the increment of the CuS nanoparticles. It can also be observed from Figure 3 that with the increase of photon energy, the absorption coefficient increases gradually and subsequently a plateau can be observed. Such increment is typical, associated to the indirect bandgap semiconductors [2]. The shifts of the absorption edge towards the lower photon energy for the nanocomposite samples denote that the optical bandgap decreased [28]. The absorption coefficient for the pure MC was found to be 5.25 eV and decreased to 2.35 eV for the doped MC with 0.08 M of CuS, as can be seen in Table 1. Such a wide shift in the absorption edge can be attributed to the formation of intramolecular charge transfer (ICT) in the host MC polymer and thus an enhancement in intermolecular stacking [29].

#### 3.1.2. Refractive Index Study

**Figure 4:**
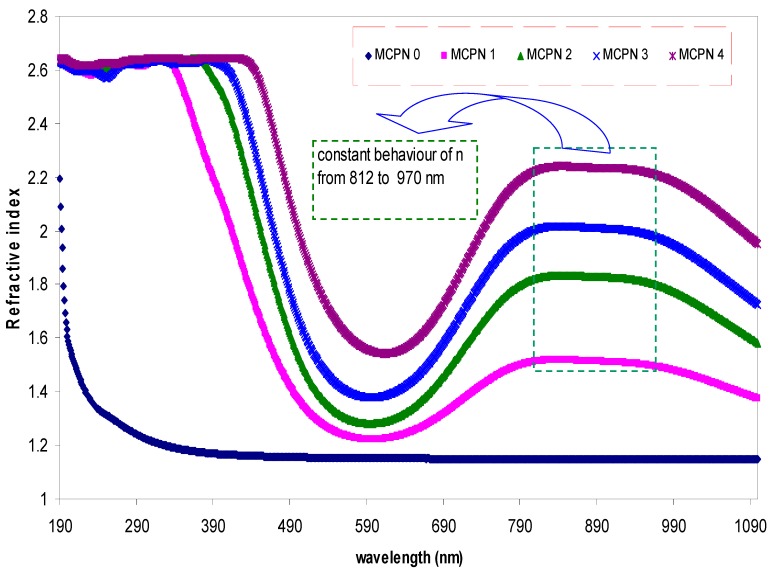
Index of refraction (n) versus wavelength for pure MC and doped MC samples.

**Figure 5:**
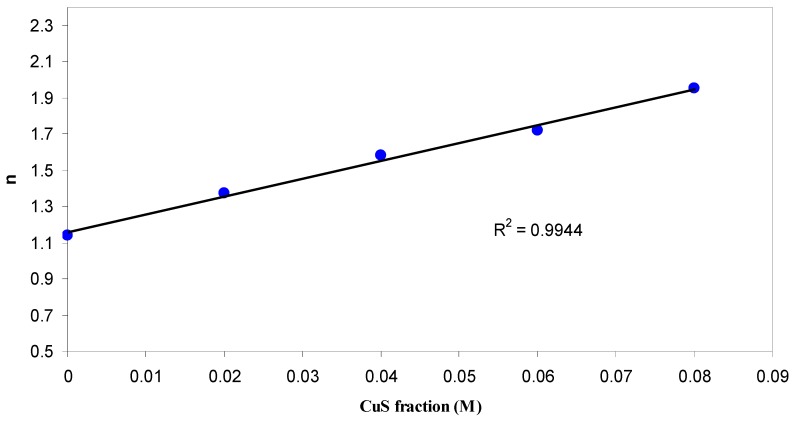
Refractive index of MC versus CuS fraction.

The refractive index examination throughout a definite range of wavelength (from near infrared to ultraviolet) is crucial for the choice of fabricated films for different applications [30]. It is believed that, from the technological viewpoint, the study of the refractive index is important in thin film investigation. From the reflection coefficient *R* and the optical extinction data, the refractive indexes can be estimated, using the Fresnel equation as follows [31]:

(2) n=(1+R1−R)+[4R(1−R)2−K2]12

where *R* is the reflection coefficient and *K =* αλ/4π is the extinction coefficient. Figure 4 shows the refractive index as a function of wavelength for the pure MC and doped MC films. It is important to notice that the doped MC samples show a distinct dispersion behavior with the wavelength, whereas the pure MC samples show an inverse relationship with the wavelength. These dispersions are essential for practical purposes since the dispersion factor plays a key role in designing optical devices [17]. The obvious peaks in the NIR region can be due to the effect of the CuS nanoparticles since they are absent in the pure MC refractive index spectra. The increase in refractive index from 1.18 to 2.06 and its shifting can be connected to the electronic structure of the doped samples. Figure 5 shows the refractive index of the samples as a function of CuS concentration. It can be seen from Figure 5 that the refractive index increases linearly with the increase of the CuS concentration. The regression *R^2^* value of 0.99 indicates a good fit to the experimental data. As experimentally and theoretically reported, the refractive index at a higher wavelength is linearly proportional to the filler concentration [32,33]. This means that the filler particles in the host polymer are well distributed [17,28]. This processing of particle distribution inside the host polymers can be regarded as an alternative method for transmission electron microscopy (TEM) investigations. From the above discussion, it is understood that the knowledge of refractive index is vital for both the particle distribution and the optical device design.

#### 3.1.3. Bandgap Study: Tauc and Optical Dielectric Methods

**Figure 6:**
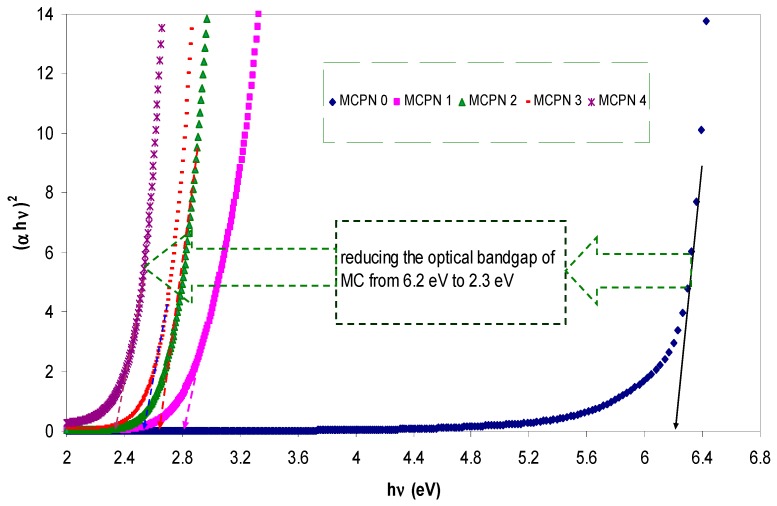
Plot of (αhυ)^2^ versus photon energy (hυ) for pure MC and doped MC films.

To properly understand and acquire information about the band structure of the solids, it is important to study and learn more about the optical absorption of the samples [34]. It is known that the fundamental absorption, which corresponds to the electron transition from the valance band to the conduction band, can be used to determine the bandgap of the material [35]. The absorption coefficient for non-crystalline materials related to the incident photon energy can be determined as given by [36]:

(3) $$\alpha =\frac{\beta }{h\upsilon }{\left(h\upsilon -E_{g}\right)}^{r}$$

where β and *E_g_* indicate a constant and the optical energy bandgap, respectively. *r* is an exponent constant, depending on the nature of the electron transitions liable to the optical absorption. The exponent *r* may have values of 1/2, 2, 3/2, and 3, corresponding to the allowed direct, allowed indirect, forbidden direct, and forbidden indirect electron excitations, respectively [35]. Figure 6 shows the plot of (α*hυ*)^2^ as a function of photon energy (*hυ*) for all the samples. The direct bandgap values were determined from the intersection of the extrapolation of the linear part of the plots to the x-axis (i.e., to the energy axis). It is established that in direct bandgap materials, the top of the valance band and the bottom of the conduction band are laid at the same zero crystal momentum (i.e., wave vector or k-vector) [37]. It is important to mention that the optical bandgap reduced from 6.2 eV for the pure MC to 2.32 eV for the doped MC with 0.08 M of CuS nanoparticles. The bandgap values for all the samples were determined, and are listed in Table 1. The optical bandgap (*E_g_*) was found to be decreased for the doped samples. Such decreases can be explained by the fact that charge transfer complexes (CTCs) in the host polymer were formed due to the incorporation of small amounts of fillers [38]. As a result, the lower energy transitions will be enhanced, leading to the observable optical bandgap changes [39]. Therefore, shifting to the visible region and observing the additional absorption peaks of the doped films are confirmations for this view. Previous studies established that organic polymers, functional materials, and composites with suitable optical bandgaps are vital for photonics, organic light-emitting diode (OLED) and optoelectronics device applications [40,41,42,43].The small optical bandgap of the MC solid nanocomposite exposes its suitability for photonics, optoelectronics, and solar cell applications. 

**Figure 7:**
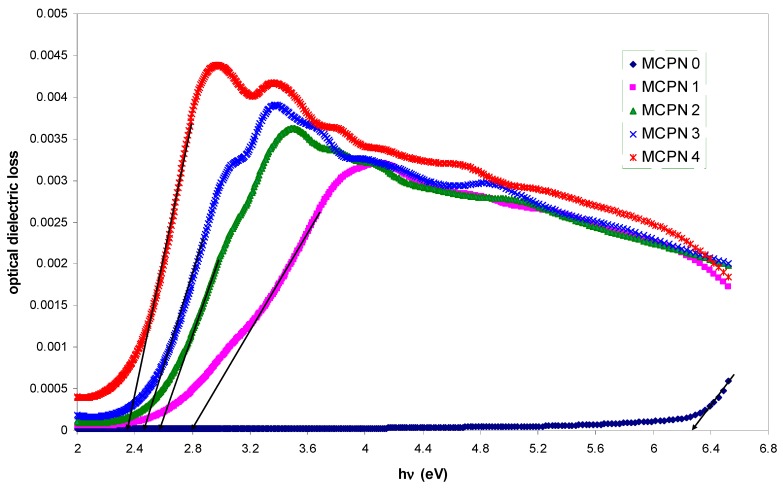
Optical dielectric loss versus photon energy (hυ) for pure MC and doped MC samples.

A better understanding of the optical properties of a solid can be gained from the study of the complex dielectric function (ɛ^*^ = ɛ_1_-i ɛ_2_), which describes the linear response of the material to electromagnetic radiation. The imaginary part ɛ_2_ stands for the optical absorption in the material, which is strongly associated with the valence (occupied) and conduction (unoccupied) bands and is given by [44]:

(4) $$\varepsilon_{2}(\omega )=\frac{2e^{2}\pi }{\Omega \varepsilon_{o}}\sum_{K,V,C}\left|\psi_{K}^{C}\right|\vec{U}\cdot \vec{r}{\left|\psi_{K}^{V}\right|}^{2}\delta \left(E_{K}^{C}-E_{K}^{V}-\hbar \omega \right)$$

where ω is the incident photon frequency, Ω is the crystal volume, e is the electron charge, ɛ_o_ is the free space permittivity, $\vec{r}$ is the position vector, $\vec{u}$ is a vector defined as the incident electromagnetic wave polarization, and $\psi_{k}^{c}$ and $\psi_{k}^{v}$ are the conduction and valence band wave functions at k, respectively. Based on the theoretical models, the optical dielectric constant is described by a complex function of frequency, which requires a large-scale computational effort to be calculated [44,45,46]. Experimentally, from the obtained refractive index and extinction coefficient data, the imaginary part of te optical dielectric function (ɛ_2_) can be estimated using the following relation [31],

ɛ_2_ = 2 *n k* (5)

where *n* is the refractive index and k is the extinction coefficient. Figure 7 shows the optical dielectric loss as a function of the applied photon energy for all the films. Here, for the pure MC film, the dielectric loss was found to be very small at low photon energy and increased exponentially at high photon energy. However, for the doped films, the absorption edge was found to be shifted to the lower photon energy and then started to decrease gradually. In addition to that, the peaks became more pronounced. It is established that the fundamental absorption edge obtained from the dielectric loss must be very close to the expected values from the Tauc’s relation [47]. This is related to the fact that the main peak shown in Figure 7 corresponds to the strong interband transitions [44]. Therefore, one can note that the obtained results from the optical bandgap of Figure 7 are in good agreement with the achieved values of Figure 6. The estimated bandgap values from both methods (i.e., Tauc and optical dielectric loss) are presented in Table 1. This work validates the fact that the optical dielectric function can be used to investigate the band structure and determine the optical bandgap [17,28]. This assumes that these parameters are directly related to the energy density of states within the optical bandgap and thus the types of electronic transitions [28,31]. In semiconductor materials, the optical bandgap and the types of the electronic transition are two important factors in the optical material identification. It is generally difficult, from the Tauc’s equation, for the nature of the electronic transition to be specified. In the present work, a very good approach which might be considered as an alternative method, has been proposed to determine the bandgap and hence the type of electronic transition can be easily specified from the Tauc’s model. From the above discussion, it is clear that the optical dielectric loss can effectively be used to study the bandgap and the Tauc’s model to determine the type of electronic transition.

**Figure 8:**
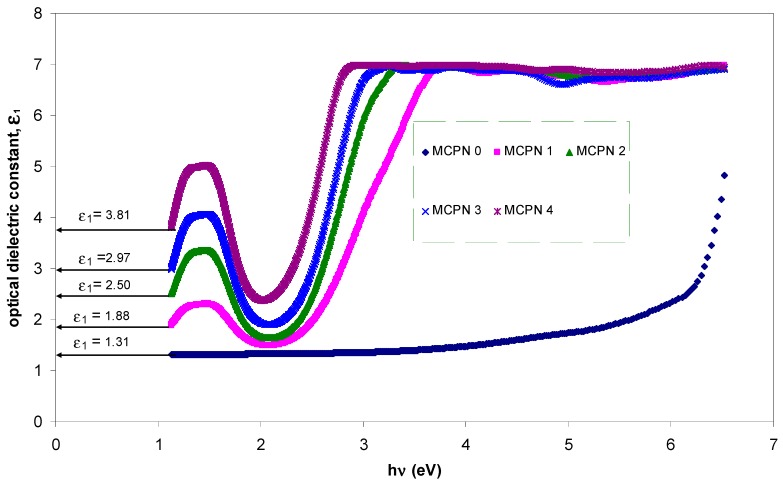
Optical dielectric constant versus photon energy for pure MC and doped MC samples.

The real part (ɛ_1_) of the dielectric function (ɛ^*^), which corresponds to the electronic part, is the most crucial physical quantity that depends strongly on the bandgap [44]. Figure 8 shows the optical dielectric constant as a function of wavelength for the pure and doped MC samples. The values of ɛ_1_ (see the inset of Figure 8) at the high wavelength (i.e., low photon energy) are increased with the square of the refractive index, which exactly meets the relation of *ɛ_1_* *= n^2^*. Furthermore, from the optical dielectric constant values, one can also anticipate the bandgap of the semiconductor materials. This can be better understood from the well-known Penn’s model [45], as given by:

(6) $$\varepsilon_{1}(0)\approx 1+{\left(\hbar \omega_{p}/E_{g}\right)}^{2}$$

**Figure 9:**
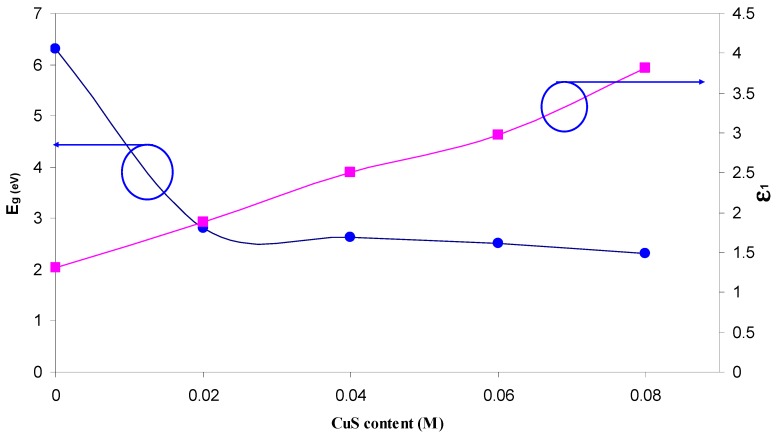
Energy bandgap and optical dielectric constant versus CuS concentration.

Equation (6) shows that a smaller energy gap (*E_g_*) yields a larger ɛ_1_ value. Figure 9 shows the plot of the energy bandgap and optical dielectric constant as a function of the filler CuS concentration. Refer to Figure 9; it is apparent that the highest value of ɛ_1_ corresponds to the smallest value of the optical bandgap. Such results reveal the validity of the Penn’s model. Therefore, the optical bandgap experimentally along with the electronic structure can be easily studied using the optical dielectric function, as shown in this work.

#### 3.1.4. Tail Study

**Figure 10:**
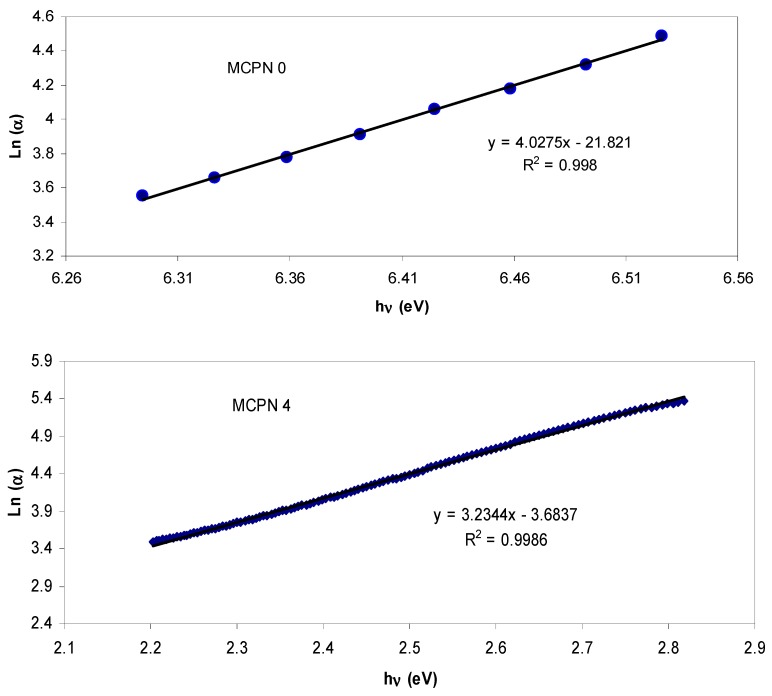
Urbach plots for pure MC (MCPN0) and doped MC (MCPN4) samples.

**Figure 11:**
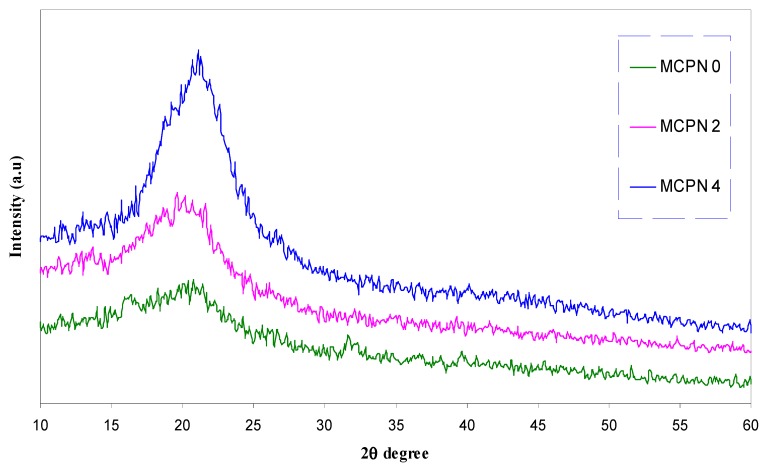
XRD pattern of pure MC and doped MC samples.

**Figure 12:**
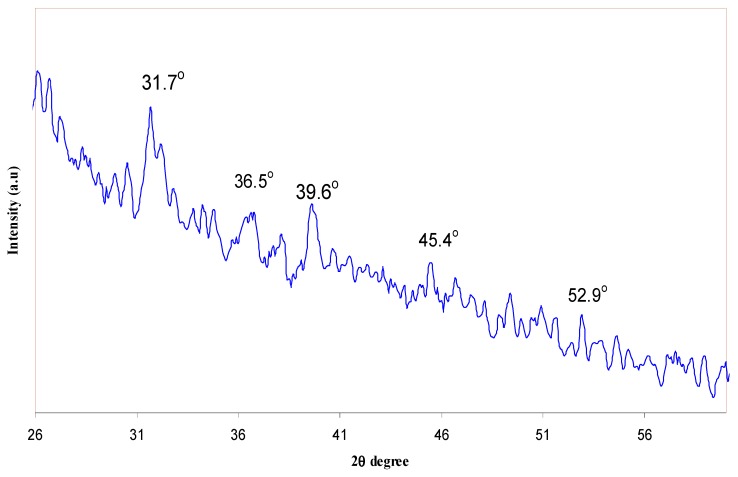
XRD pattern for MCPN4 sample after smoothing.

Figure 10 shows the Urbach plot of the pure and doped MC (MCPN 4) samples. In amorphous materials, it has been suggested that the tailing of the density of states in the forbidden energy gap can be attributed to the lack of long-range order [38]. The photon absorption will be related to the existence of localized tail states in the forbidden gap. The width of this tail, named as the Urbach tail, indicates the defect levels in the gap. The width of the Urbach tail can be calculated by [48],

α = α_o_·exp(*h*ω/*E_u_*) (7)

where α*_o_* is a constant and *E_u_* refers to the energy of the band tail or is sometimes known as the Urbach energy [49]. The value of the Urbach energy inversely varies with the optical bandgap. It is reported that an increase in the structural disorder in polymer composites can be assigned to the increase of *E_u_*. Consequently, the states, from band to tail, will be redistributed and hence it enables a greater number of possible transitions from bands to tail and tail to tail [50]. On the other hand, a decrease in optical bandgap can be achieved. The calculated Urbach energy was found to be 248 meV for the pure MC sample and it increased to 309 meV for the doped MC sample with 0.08 M of the CuS nanoparticle. Previous reports confirmed that the increase of the crystalline region in the sample can be an indication of the decrease of the Urbach energy, whereas its increase is indirectly associated to an increased amorphous portion [28,49,51,52]. Figure 11 shows the XRD pattern of the pure MC and doped MC samples. It is well reported that a broad crystalline hump at around 2θ = 19–21° appears to correspond to the intermolecular hydrogen bonding along with a short-distance order for the sample chains in the MC polymer [53,54]. As one can see in Figure 11, for 0.04 M and 0.08 M of the CuS filler samples, the intensity significantly decreases and the peak broadening increases. This can be attributed to the fact that the synthesized CuS nanoparticles have disrupted the crystalline nature of the host polymer [54]. The XRD results indicate the increase of the amorphous fraction in the nanocomposite samples, i.e., the disorder is dominant in the doped samples. Therefore, the results of XRD observed in this work can support the Urbach energy (*E_u_*). It is understood that Urbach energy can be used to investigate the order and disorder behavior of the materials. From the XRD pattern observed for the MCPN4 sample, some peaks exhibit low intensity. In order to further distinguish such peaks, smoothing has been carried out. Figure 12 shows the XRD pattern after smoothing within the scanning ranges of 26° ≤ 2θ ≤ 60°. The peaks appeared at 2θ = 31.7°, 36.5°, 39.6°, 45.4°, and 52.9°, which are ascribed to the existence of the CuS nanoparticles [17]. On the other hand, a significant change in FTIR bands as can be seen in the later sections is evidence for a complex formation between the MC polymer and the CuS nanoparticles.

### 3.2. FTIR Study

**Figure 13:**
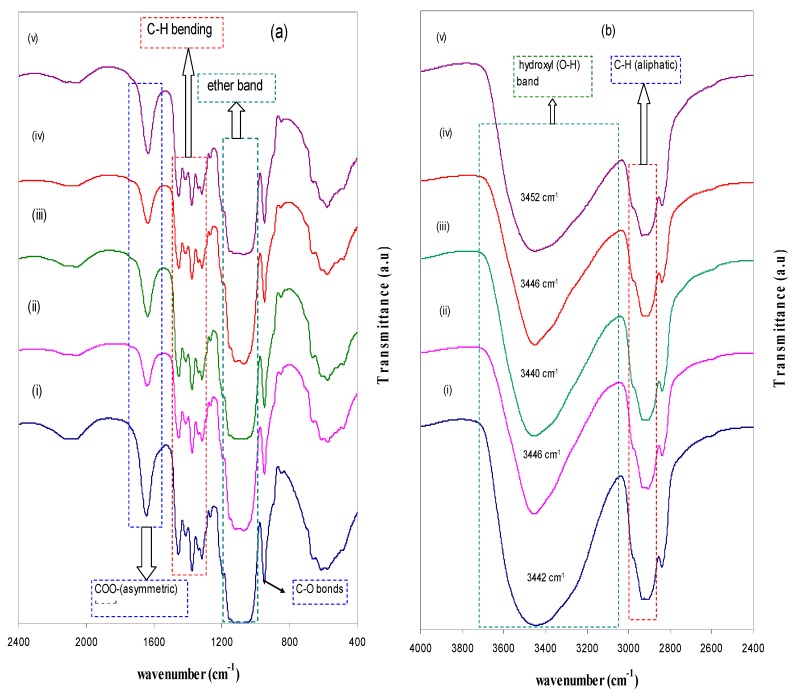
FTIR spectra of (i) MCPN0 (pure MC), (ii) MCPN1, (iii) MCPN2, (iv) MCPN3, and MCPN4 in the region (**a**) 400 to 2400 cm^−1^ and (**b**) 2400 to 4000 cm^−1^.

Figure 13a,b shows the FTIR spectra of the pure MC and doped MC films in the regions of (a) 400 cm^−1^ to 2400 cm^−1^ and (b) 2400 cm^−1^ to 4000 cm^−1^. Hydrogen bond formation in the nanocomposite based MC films can be understood by IR spectroscopy since hydrogen bonding changes the stretching vibration frequencies [55]. The hydroxyl band observed for the pure MC can be ascribed to the wavenumber region 3447–3458 cm^−1^, though the ether band needs to be at 1066 cm^−1^ and 1110 cm^−1^ [56]. The absorption bands that appeared at 3458 cm^−1^ in the pure MC and doped MC samples can be ascribed to the O-H stretching [57,58]. The bands at 1422, 1608, and 2931 cm^−1^ are assigned to the stretching vibration of COO– (symmetric), COO– (asymmetric), and C–H (aliphatic), respectively [57]. Small absorption bands in the range between 1251 and 1455 cm^−1^ are considered to be the characteristics of the C–H bending of MC. Small bands in the range of 487–654 cm^−1^ were observed due to C–H vibrations [58]. The free (or unbounded) hydroxyl group absorbs strongly in the region 3650–3500 cm^−1^. The absence of these bands reveals that most of the hydroxyl groups are bounded and as a result, shifting can be observed as depicted in Figure 4b. The existence of the bound hydroxyl group may induce a shift of the absorption to a smaller frequency. Consequently, the intensity will be increased and an asymmetrical peak will appear. Along with that, the band will also be broadened. The hydroxyl absorption is a measure of strength and interactions of the hydrogen bond in the polymer matrix [55]. It is evident from Figure 4b that the hydroxyl band for the pure MC has appeared as a symmetric and homogeneous shoulder, whereas a broad and asymmetric shoulder can be seen for the nanocomposite samples. As is clear from the figure, the range from 950 to 1250 cm^−1^ is the most informative range. Here, a complex strong absorption band profile of methylcellulose is observed, which is mainly due to the C–O bonds’ stretching vibrations in the cellulose IR spectra and its ethers [59]. The intensity of the C–O bonds is also found to be decreased for the doped samples. This can be evidence that the MC host polymer and synthesized nanoparticles have a strong interaction. The significant decrease of transmittance intensity and bands’ shifting reveal the process of adsorbed nanoparticles on the MC polymer functional groups and formation of the charge transfer complexes. The adhesion of CuS nanoparticles may reduce the vibration of the polar groups [60].

## 4. Conclusions

In this work, polymer nanocomposites based on methyl cellulose (MC) with a small optical bandgap have been prepared, using in situ techniques. The transmittance spectra for the nanocomposite samples have been recorded and displayed a transparency of above 80%. Distinguishable peaks were observed in the wavelength range from 680–1090 nm, which can be ascribed to the surface resonance plasmonic (SRP) peaks of the CuS nanoparticles. A wide shift of the absorption edge to lower photon energies has been found to be related to the formation of the charge transfer complex in the MC host polymer. The refractive index spectrum of the nanocomposite samples exhibited a clear dispersion. The linear relationship between the refractive index and CuS fraction was also examined and indicated a good dispersion of the nanoparticles in the host polymer. The optical bandgap of MC was found to be reduced from 6.2 to 2.3 eV for the doped MC sample with 0.08 M of CuS nanoparticles. Such a reduction confirms the formation of the charge transfer complex between the polymer chains and thus introduces many trap sites between the valence and conduction bands. In this study, the optical dielectric loss was precisely used to estimate the optical bandgap, whereas, the Tauc’s method was used to identify the type of electronic transition. From the plot of the optical dielectric constant versus CuS concentration, the highest dielectric constant associating with the smallest optical bandgap has been observed. This noticeable result revealed the validity of Penn model. Moreover, the Urbach energy for the nanocomposite samples was found to be increased, which was considered as a confirmation of a great number of possible bands-to-tail and tail-to-tail transitions. The broad nature of the XRD pattern of the doped samples compared to the pure MC revealed the higher amorphous fraction in the doped samples. The increase of the Urbach energy from 248 meV for the pure MC sample to 309 meV for the doped MC sample with 0.08 M of CuS nanoparticle supports the enhancement in the amorphous fraction. The appearance of some peaks in the XRD pattern of the MC nanocomposites confirms the formation of the CuS crystalline nanoparticles. Finally, the significant decrease of the transmittance intensity and bands’ shifting within the FTIR spectra for the doped samples support the complex formation between the MC host polymer and the distributed CuS nanoparticles.

## Figures and Tables

**Table 1:** Absorption edge and optical bandgaps for MC and doped MC samples.

Nano-Composite Designation	Absorption Edge (eV)	Indirect Bandgap (eV)	Estimated Bandgap from ɛ"-*hυ* Plot
MCPN0	6.2	6.19	6.24
MCPN1	2.8	2.78	2.8
MCPN2	2.5	2.6	2.6
MCPN3	2.4	2.47	2.48
MCPN4	2.28	2.33	2.35
